# Input estimation for drug discovery using optimal control and Markov chain Monte Carlo approaches

**DOI:** 10.1007/s10928-016-9467-z

**Published:** 2016-03-01

**Authors:** Magnus Trägårdh, Michael J. Chappell, Andrea Ahnmark, Daniel Lindén, Neil D. Evans, Peter Gennemark

**Affiliations:** University of Warwick, School of Engineering, Coventry, CV4 7AL UK; CVMD iMed DMPK, AstraZeneca R&D, 431 83 Mölndal, Sweden; CVMD iMed Bioscience, AstraZeneca R&D, 431 83 Mölndal, Sweden

**Keywords:** Input estimation, Deconvolution, Nonlinear dynamic systems, Optimal control, Markov Chain Monte Carlo method

## Abstract

Input estimation is employed in cases where it is desirable to recover the form of an input function which cannot be directly observed and for which there is no model for the generating process. In pharmacokinetic and pharmacodynamic modelling, input estimation in linear systems (deconvolution) is well established, while the nonlinear case is largely unexplored. In this paper, a rigorous definition of the input-estimation problem is given, and the choices involved in terms of modelling assumptions and estimation algorithms are discussed. In particular, the paper covers *Maximum a Posteriori* estimates using techniques from optimal control theory, and full Bayesian estimation using Markov Chain Monte Carlo (MCMC) approaches. These techniques are implemented using the optimisation software CasADi, and applied to two example problems: one where the oral absorption rate and bioavailability of the drug eflornithine are estimated using pharmacokinetic data from rats, and one where energy intake is estimated from body-mass measurements of mice exposed to monoclonal antibodies targeting the fibroblast growth factor receptor (FGFR) 1c. The results from the analysis are used to highlight the strengths and weaknesses of the methods used when applied to sparsely sampled data. The presented methods for optimal control are fast and robust, and can be recommended for use in drug discovery. The MCMC-based methods can have long running times and require more expertise from the user. The rigorous definition together with the illustrative examples and suggestions for software serve as a highly promising starting point for application of input-estimation methods to problems in drug discovery.

**Electronic supplementary material:**

The online version of this article (doi:10.1007/s10928-016-9467-z) contains supplementary material, which is available to authorized users.

## Introduction

**Fig. 1:**
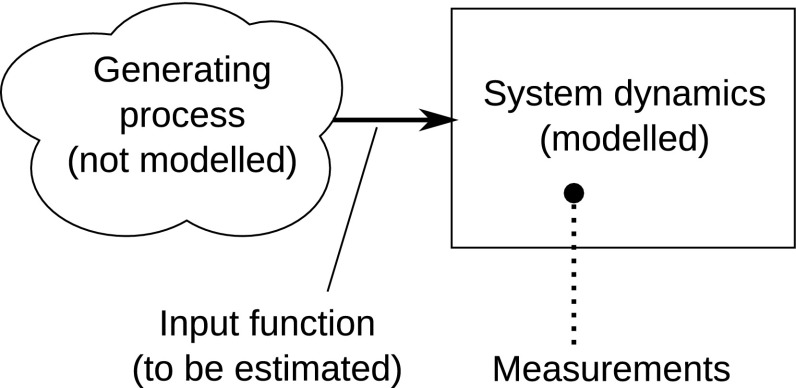
The input estimation problem: given measurements and known system dynamics, estimate the input function without modelling its generating process

Pharmacokinetic and pharmacodynamic (PKPD) models are generally represented using a system of ordinary differential equations (ODEs). The structure and parameter values are usually estimated from experimental data. In some cases, it is useful to estimate the time-course of a variable even when no generating model is available. This can be done using experimental data combined with a model connecting the sought variable with the measurements. This paper addresses one such case: estimating the unknown input function to a known dynamical system, given sparse measurements of certain state variables (Fig. 1).

A typical example is to estimate the oral absorption-rate of a drug, given measurements of the drug plasma concentration. This assumes that a model of drug distribution and elimination is available. Of particular interest is the *oral bioavailability*—the fraction of the drug that is absorbed [22]. Clearly, this also applies to other routes of administration, e.g. subcutaneous. Another example is to estimate the energy intake of a subject given body-mass measurements. These estimates are important in research on drugs aimed at reducing body mass, or improving metabolic parameters, or both [11, 15].

One possible approach is to assume that the input function has a prespecified functional form, parametrised with a small number of parameters. Examples of functions used for this purpose include exponentials [37] and inverse Gaussians [7]. In this paper, we consider non-parametric methods that do not make strong assumptions about the form of the input.

When the dynamics are linear, established estimation methods exist [8, 37]. These methods rely on being able to express the input–output relationships as convolution integrals, something that is possible for linear systems only. Additionally, in many cases the linear input-estimation problem can be reduced to solving a quadratic optimisation problem [37]. In contrast, estimation in nonlinear systems is a more difficult problem. While many potentially useful methods exist in the engineering and statistical literature, typical engineering applications have densely sampled data, and these methods are not necessarily a good fit for PKPD applications. Thus, their applicability needs to be assessed on a case-by-case basis.

In this paper, we give a rigorous definition of the input-estimation problem for nonlinear systems, and suggest methods and software to solve the problem. Two case studies are presented, using different choices of algorithms to illustrate the methods and serve as a starting point for discussions.

## Problem specification

We consider models of the form:

1 $$\begin{aligned} \frac{{\mathrm {d}}\mathbf {x}(t)}{{\mathrm {d}}t} \,= \;& {} \mathbf {f}(t, \mathbf {x}(t), \mathbf {u}(t)) \end{aligned}$$

2 $$\begin{aligned} \mathbf {y}(t)\,=\; & {} \mathbf {g}(t, \mathbf {x}(t), \mathbf {u}(t), \mathbf {v}(t)) \end{aligned}$$

3 $$\begin{aligned} \mathbf {x}(t_0)\,= \;& {} \mathbf {x}_0 \end{aligned}$$

where $t \in \mathbb {R}$ is time, $\mathbf {x}(t) \in \mathbb {R}^{d_x}$ is the system state, $\mathbf {u}(t) \in \mathbb {R}^{d_u}$ is the input function to be estimated, $\mathbf {y}(t) \in \mathbb {R}^{d_y}$ are the measured quantities, $t_0 \in \mathbb {R}$ is the initial time and $\mathbf {x}_0 \in \mathbb {R}^{d_x}$ is the initial state. The function $\mathbf {f} : \mathbb {R} \times \mathbb {R}^{d_x} \times \mathbb {R}^{d_u} \mapsto \mathbb {R}^{d_x}$ is the right-hand side of the system of ODEs, and $\mathbf {g} : \mathbb {R} \times \mathbb {R}^{d_x} \times \mathbb {R}^{d_u} \times \mathbb {R}^{d_v} \mapsto \mathbb {R}^{d_y}$ is the measurement equation, which has an extra variable $\mathbf {v}(t) \in \mathbb {R}^{d_v}$ representing the measurement noise. The dimensionalities $d_x$, $d_u$, $d_y$ and $d_v$ can take any integer values. The functions $\mathbf {x}(t)$, $\mathbf {u}(t)$, $\mathbf {y}(t)$ and $\mathbf {v}(t)$ may have constraints, for example to exclude negative values. $\mathbf {f}(\cdot )$ is assumed to satisfy the technical requirements for the system of ODEs to have a unique solution. No further assumptions on $\mathbf {f}(\cdot )$ and $\mathbf {g}(\cdot )$ are made, although in practice, some estimation methods may have additional requirements such as differentiability. In particular, $\mathbf {f}(\cdot )$ and $\mathbf {g}(\cdot )$ can be nonlinear. If they are linear, standard methods exist for input estimation [8, 37]. The measured quantities $\mathbf {y}(t)$ are sampled at a finite set of time points $t_i$, $i \in \{1, \ldots , n\}$. The set of sampled measurements is denoted $\mathbf {y}_{1:n}$.

The aim is to estimate $\mathbf {u}(t)$ given a set of measurements, $\mathbf {y}_{1:n}$. To make this problem well-posed, it is necessary to impose additional assumptions on the input function. To see this, consider a maximum likelihood estimator, that gives the estimated input ${{\hat{\varvec{u}}}}(t)$ as:

4 $$\begin{aligned} {{\hat{\varvec{u}}}}(t) =\mathop {{\mathrm {argmin}}}\limits _{\mathbf {u}(t)} E_D \end{aligned}$$

where $E_D$ is the negative log likelihood function. Since $\mathbf {u}(t)$ is an arbitrary function, and therefore infinite-dimensional, it cannot be uniquely determined from a finite set of measurements. This can be remedied by adding a regularisation term $E_W$, that penalises unnecessarily complex solutions. The estimator can now be written as:

5 $$\begin{aligned} {{\hat{\varvec{u}}}}(t) = \mathop {{\mathrm {argmin}}}\limits _{\mathbf {u}(t)} \left( E_D + \tau E_W \right) \end{aligned}$$

where $\tau$ is a *regularisation parameter* that controls the trade-off between data fit and regularity [37].

This can also be interpreted as a Bayesian inference problem, where the aim is to determine the posterior distribution:

6 $$\begin{aligned} p(\mathbf {u}(t)|\mathbf {y}_{1:n}) =\; \frac{p(\mathbf {y}_{1:n}|\mathbf {u}(t))p(\mathbf {u}(t))}{p(\mathbf {y}_{1:n})} \end{aligned}$$

where $p(\mathbf {y_{1:n}}|\mathbf {u}(t))$ is the likelihood, $p(\mathbf {u}(t))$ is the prior, and $p(\mathbf {y}_{1:n})$ is the model evidence, which is constant for a given dataset. By identifying $\ln p(\mathbf {y}_{1:n}|\mathbf {u}(t)) = -E_D$ and $\ln p(\mathbf {u}(t)) = -\tau E_W$, it can be seen that Eq. (5) gives the *Maximum a Posteriori* (MAP) estimate. An advantage of the Bayesian interpretation is that it is possible to calculate other statistical quantities of interest, such as mean or median estimates as well as credible intervals.

Together with the model and the data, the problem can be specified by the following components:

- Choice of prior for $\mathbf {u}(t)$, or equivalently, choice of regularisation term. This encodes the prior assumptions about the shape of the input function.
- Functional representation. In practice, the input function $\mathbf {u}(t)$ has to be represented using a finite set of parameters. Therefore, a choice of basis for the input function must be made.
- Desired statistical quantities. Is a MAP estimate enough or are other quantities such as credible intervals desired?

We will now describe these three components in more detail.

### Choice of prior for $\mathbf {u}(t)$

In this section, priors for scalar input functions are discussed. For vector-valued input functions, priors can be independently assigned to each component, as long as they are assumed to be *a priori* independent. Assigning priors jointly over all components is not discussed in this paper.

For scalar input functions *u*(*t*), a common choice of prior is to penalise the first or second derivative of the function in order to avoid unnecessarily oscillatory functions [37, p. 87]. If the function is defined in the interval [*a*, *b*], the unnormalised log prior becomes

7 $$\begin{aligned} \ln p(u(t)) \propto - \tau \int _a^{b} \left( \frac{{\mathrm {d}}^j u}{{\mathrm {d}}t^j} \right) ^2 {\mathrm {d}}t \end{aligned}$$

Typically, here *j* is set to 1 or 2.

In a Bayesian setting, the prior penalising the *j*th derivative can be interpreted as defining a special case of a Gaussian process [33]. This is a stochastic process whose finite-dimensional marginal distributions are Gaussian, and it is completely determined by a mean and a covariance function. The theory of Gaussian processes provides a flexible framework for defining priors over functions. However, this approach does not seem to have been employed in drug discovery modelling to any significant extent.

Another choice for scalar functions is to use the entropy of a discrete-time version of the input $u_k$ with $N_e$ steps:

8 $$\begin{aligned} \ln p(u(t)) \propto -\tau \sum _{k=0}^{N_e-1} u_k \ln \frac{u_k}{m_k} \end{aligned}$$

where $m_k$ is a baseline value that can be set to the average of the nearest neighbours [20]. Conceptually, this approach prefers functions where the function’s value at each time point is similar to its neighbours.

### Functional representation of $\mathbf {u}(t)$

For notational convenience, this section discusses scalar-valued functions *u*(*t*) only. When the input function is vector-valued, each component can be represented using the techniques described below.

To represent the input function using a finite set of parameters, one can select a set of basis functions $B_i(t)$ and write the input function as a linear combination of these:

9 $$\begin{aligned} u(t) = \sum _{i=0}^{N_B-1} \theta _i B_i(t) \end{aligned}$$

Once a choice of basis has been made, recovering the input function is equivalent to recovering the coefficients $\theta _i$.

Perhaps the simplest choice of basis is to represent the function as a piecewise constant function, as done in [29]. These functions are very cheap to evaluate. On the other hand, the resulting staircase-like functions have to be defined on a relatively dense grid to represent the actual function well. This makes for a high-dimensional estimation problem which can cause computational difficulties.

A convenient basis for a zero-mean Gaussian process *u*(*t*) is given by the Karhunen–Loève expansion [24]. This method computes a set of basis functions such that their coefficients will be independent zero-mean Gaussian random variables. A good approximation can be obtained by only retaining the basis functions contributing the most to the input function. This can be viewed as a dimensionality-reduction method. The fact that the coefficients are uncorrelated can make it easier to efficiently use sampling-based estimation methods. More formally, assume that the process is defined in the interval [*a*, *b*] and has a covariance function *k*(*s*, *t*). If the functions $\phi _i(t)$ and values $\lambda _i$ satisfy the eigenvalue problem:

10 $$\begin{aligned} \int _a^b k(s, t)\phi _i(s)\,{\mathrm {d}}s = \lambda _i \phi _i(t) \end{aligned}$$

then the process can be represented by:

11 $$\begin{aligned} u(t) = \sum _{i=0}^{\infty } \theta _i \sqrt{\lambda _i} \phi _i(t) \end{aligned}$$

where the coefficients $\theta _i$ are independent zero-mean Gaussian random variables with unit variance. By setting $B_i(t) = \sqrt{\lambda _i}\phi _i(t)$ and keeping the basis functions with the $N_B$ largest eigenvalues, the prior can be placed on a finite number of coefficients rather than on the function itself, effectively transforming the input estimation problem to a parameter estimation problem.

In [28], the Karhunen–Loève expansion is given for penalisation of the first and second derivatives of the input function, and applied to determining the input to a linear system. For these priors, the basis functions do not depend on the regularisation parameter. This is important since the regularisation parameter is usually unknown and has to be estimated from the data.

The Karhunen–Loève expansion assumes that the input function starts at 0. When penalising the second derivative, the derivative too starts at 0. To allow functions to start at arbitrary values, a constant or a linear term can be added.

Alternatively, splines can be used [5]. Just as piecewise constant functions, they are non-zero only in a limited interval. They can be computed efficiently, can be made differentiable to an arbitrary degree, and can represent realistic-looking functions using relatively few parameters.

### Desired statistical quantities

In classical regularisation theory, the quantity of interest is the penalised maximum likelihood, given by Eq. (5). From a Bayesian perspective, this is the MAP estimate. One might also be interested in the mean input function, as well as pointwise 95 % credible intervals. The latter is important for determining the uncertainty of the estimate.

## Estimation algorithms

MAP estimates can be obtained by optimising Eq. (5). Algorithms for optimisation in dynamical systems are studied in the field of *optimal control*.

When quantities other than MAP are of interest, inference methods such as Markov Chain Monte Carlo (MCMC) approaches can be employed [6]. Although not explored here, it would also be possible to use other sampling methods such as Sequential Monte Carlo [9] or analytical approximations such as Variational Bayesian methods [3].

Another estimation decision is how to select the regularisation parameter $\tau$. The *discrepancy criterion* suggests selecting $\tau$ so that the sum of squared residuals is equal to the expected sum of squared distances between the true function and the measurements. In *ordinary and generalised cross-validation*, measurements are left out from the estimation procedure, and the ability to predict these left-out measurements is assessed. For linear Gaussian problems, it is possible to derive analytical maximum-likelihood criteria for $\tau$ [35]. In the *L-curve approach* [18], a MAP estimate is calculated for a large number of values for $\tau$, and the data and regularisation cost terms $E_D$ and $E_W$ are plotted against each other. For a low $\tau$, the data fit will be almost perfect and the data cost will be almost zero. Conversely, for high $\tau$, the input function is forced to follow the regularisation criterion, and the regularisation cost will approach a minimum value. Between these extremes, there is a characteristic corner in the plot, where there is a reasonable trade-off between data fit and regularity. In the Bayesian paradigm, $\tau$ can be treated as an additional parameter, and can be estimated together with the basis function coefficients.

### Optimal control-based methods

The aim of optimal control is to select the input to a dynamical system that minimises some cost function. Here, the cost function is the negative log posterior. The optimisation problem can be formulated in multiple ways [4, 32].

In *single shooting*, only the parameters describing the input function are included as decision variables in the optimisation problem. The log posterior for a given input function is calculated by solving the system of differential equations using a numerical ODE solver. This is the most straightforward method.

In *multiple shooting*, the time course of the system is divided into a number of sub-intervals. For each such sub-interval, the system of ODEs is solved numerically. The input function parameters as well as the state variables at the start of each interval are included as decision variables. Constraints are added to the problem to ensure that the resulting trajectories are continuous. This results in an optimisation problem that is larger than in single shooting, but it tends to be sparser and less non-linear.

In *collocation methods*, no ODE solvers are used. Instead, the dynamic model is included in the form of equality constraints in the optimisation problem. The time course of the system is divided into a number of sub-intervals. In each sub-interval, the state trajectories are approximated by low-order polynomials. A small number of *collocation points* are selected in each interval, and constraints are added to ensure that the solution of the system of ODEs is satisfied at these points. The input function parameters as well as the state variables at the start of each interval and at all collocation points are included as decision variables. Additional constraints are added to ensure that the state trajectory is continuous. This results in an even larger optimisation problem than multiple shooting, but it tends to be even sparser and less non-linear.

Many optimisation methods rely on gradients and Hessians of the objective function. A straightforward way to compute these is to use finite differences. However, this can be inaccurate and slow, especially in high dimensions. A powerful alternative is to use *automatic differentiation*, where gradients are automatically computed by applying the chain rule of calculus to the objective function [31]. For numerical ODE solvers, it is also possible to solve the *sensitivity equations* to obtain gradients [2]. For input-estimation problems, a combination of these two methods can be used.

An advantage of optimal control-based methods is that they can be very fast, even for high-dimensional functional representations such as piecewise constant functions on a dense grid. It is easy to include extra constraints, which is helpful in avoiding unphysical answers, such as negative concentrations. The main disadvantage is that they can only provide MAP estimates. It is therefore hard to assess the uncertainty of the estimate.

### Markov chain Monte Carlo (MCMC)

Most quantities of interest in Bayesian inference cannot be computed in closed form. One way to overcome this is to use Monte Carlo methods [34]. Let $\varvec{\theta }$ be the vector of parameters determining the function $\mathbf {u}(t)$, which can include the basis function coefficients, the regularisation parameter, and other parameters. The idea is that the posterior $p(\varvec{\theta }|\mathbf {y})$ can be approximated by a large number of samples $\varvec{\theta }^{(i)}$ drawn from it. This can, somewhat informally, be represented by:

12 $$\begin{aligned} p(\varvec{\theta }|\mathbf {y}) \approx \frac{1}{N}\sum _{i=0}^{N-1} \delta _{\varvec{\theta }^{(i)}}, \quad \varvec{\theta }^{(i)} \sim p(\varvec{\theta }|\mathbf {y}) \end{aligned}$$

where $\delta _{\varvec{\theta }^{(i)}}$ is the Dirac function centred on $\varvec{\theta }^{(i)}$. Using these samples, any quantity of interest can be calculated by simple arithmetic. However, drawing samples from the posterior is in itself a non-trivial problem. Markov Chain Monte Carlo methods solve this by constructing a Markov chain such that its samples marginally come from the correct distribution. The Metropolis–Hastings algorithm is one of the most general MCMC methods [19, 27]. At each time index *i*, a new sample $\varvec{\theta }^{(i)}$ is drawn using the following algorithm:

1. Propose a new sample $\varvec{\theta }^\prime$ using an arbitrary proposal distribution $p(\varvec{\theta }^\prime |\varvec{\theta }^{(i-1)})$.
2. Calculate the Metropolis–Hastings ratio *A*:
  $$\begin{aligned} A = \min \left( 1, \frac{p(\varvec{\theta }^\prime |\mathbf {y})p(\varvec{\theta }^{(i-1)}|\varvec{\theta }^\prime )}{p(\varvec{\theta }^{(i-1)}|\mathbf {y})p(\varvec{\theta }^\prime |\varvec{\theta }^{(i-1)})} \right) \end{aligned}$$
3. With probability *A*, set $\varvec{\theta }^{(i)} = \varvec{\theta }^\prime$. Otherwise, set $\varvec{\theta }^{(i)} = \varvec{\theta }^{(i-1)}$.

It can be shown that the resulting sequence $\varvec{\theta }^{(i)}$ will asymptotically be marginally distributed according to $p(\varvec{\theta }|\mathbf {y})$. Most MCMC methods in use can be shown to be special cases of the Metropolis–Hastings algorithm. The parameters can be updated either one at a time, or several at once. For a good introduction to MCMC, see [6] and [13].

In some cases, it is possible to sample from some of the parameters conditional on the other ones. This typically happens when *conjugate priors* are used, which are priors that have the same functional form as their corresponding posteriors. As an example, when Karhunen–Loève basis functions are used, their coefficients are normally distributed with a precision equal to the regularisation parameter. If the regularisation parameter is assigned a Gamma distribution prior, it can be shown that its distribution conditioned on the coefficients of the basis functions is also a Gamma distribution. Since efficient methods exist for sampling from Gamma and other standard distributions, this can be used to propose new parameter values. By inserting this proposal into the Metropolis–Hastings ratio, it can be shown that these proposals will always be accepted. This sampling method is called *Gibbs sampling* [6].

MCMC methods have the advantage that they can handle arbitrary models and estimate any kind of statistical quantity, including credible intervals. The downside is that they can be slow, since a large number of samples may have to be generated. Their performance can depend critically on the choice of proposal distribution. For high-dimensional functional representations, finding a proposal distribution that gives acceptable performance can sometimes be challenging.

One possible approach to construct good proposal distributions is to use Riemannian manifold methods [14]. In these methods, a *metric tensor* is defined that describes the local geometry of the target distribution. This can be used to construct proposals that automatically make larger steps in the directions for which the distribution changes slowly. In [14], it is suggested to use the sum of the Fisher information matrix and the negative Hessian of the log prior as a metric tensor. The simplest manifold method is the Simplified Manifold Metropolis-Adjusted Langevin Algorithm (SMMALA). In SMMALA, the new sample $\varvec{\theta }^{\prime }$ is proposed from a Gaussian distribution with mean $\varvec{\mu }$ and covariance $\mathbf {C}$:

13 $$\begin{aligned} \varvec{\mu }\,=\, & {} \varvec{\theta }^{(i-1)} +\frac{1}{2}\epsilon ^2 \mathbf {G}^{-1}\left( \varvec{\theta }^{(i-1)}\right) \frac{{\mathrm {d}} \left( \ln p(\varvec{\theta }|\mathbf {y})\right) }{{\mathrm {d}}\varvec{\theta }} \bigg |_{{\varvec{\theta }}=\varvec{\theta }^{(i-1)}} \end{aligned}$$

14 $$\begin{aligned} \mathbf {C}\,=\, & {} \epsilon ^2 \mathbf {G}^{-1}\left( \varvec{\theta }^{(i-1)}\right) \end{aligned}$$

where $\mathbf {G}(\cdot )$ is the metric tensor and $\epsilon$ is a user-specified scale factor. An intuitive motivation for SMMALA is that it proposes values from a quadratic approximation of the log target distribution. The required gradients can be obtained by using automatic differentiation and solving the corresponding sensitivity equations.

It is also possible to mix proposals, for example by using different updating mechanisms for different parameters. This is exemplified here in Case Study 2.

Even though the samples from the Markov chain are drawn from the desired distribution asymptotically, the initial part of the chain may be non-representative and should be discarded. Various methods have been proposed to assess whether convergence to the desired distribution has been achieved [25]. In *Geweke’s method*, the mean and variance from different segments of the chain are compared [12]. The *Gelman–Rubin method* compares the within-chain and between-chain variance of several Markov chains initialised in different parts of the parameter space [10]. In the *Raftery–Lewis method*, the user can specify that quantiles of particular parameters should be estimated to a given accuracy. By analysing statistics of where the parameters exceed these quantiles, an estimate of the number of samples to discard can be obtained [30].

Assessing the number of samples required for an accurate estimate is non-trivial. Since the samples generated by the Markov chain are correlated, *N* samples from the chain give a less accurate estimate than *N* independent samples. The previously mentioned Raftery–Lewis method can provide an estimate for the required number of samples. The *effective sample size* (ESS) is another way to assess the quality of the samples. It is a rough estimate of the number of independent samples required to obtain the same approximation error as the samples from the Markov chain and can be calculated from the autocorrelation of the generated samples [14, Sect. 7.1].

## Previous work

Many previously used nonparametric input-estimation methods can be described using the framework presented above:

Verotta [37] gives a good overview of classical input-estimation (deconvolution) methods for linear systems. As a choice of prior, two approaches are suggested: either using the norm of the first or second derivative of the input function, or parametrising the input function with few enough parameters so that the problem becomes well-posed without the use of a prior. As basis functions, piecewise constant and spline functions are suggested. Obtaining point estimates using optimisation techniques is discussed. Suggested methods to assess uncertainty estimates include quadratic approximations around the MAP estimate and bootstrapping. The methods are tested on pharmacokinetic examples as well as an example involving estimating the secretion rate of lutenizing hormone.

Magni et al. [26] employ MCMC techniques to do full Bayesian inference using piecewise constant basis functions and a prior penalising the first or second derivative, and use this to estimate insulin secretion rate after a glucose stimulus.

Pillonetto et al. [29] suggest penalising the first derivative of the logarithm of the input function to handle non-negativity constraints. Piecewise constant basis functions are used, and full inference is done using MCMC. The method is applied to estimate lutenising hormone secretion rate as well as to pharmacokinetic problems.

Pillonetto and Bell [28] suggest using Karhunen–Loève basis functions with various priors. Since their examples are unconstrained linear Gaussian models, full inference can be done analytically without resorting to sampling methods. However, MCMC is used to estimate the regularisation parameter. The methods are tested on synthetic test functions.

Hattersley et al. [20] use an entropic prior together with piecewise constant basis functions. MAP estimates are obtained using a Sequential Quadratic Programming optimisation method. The method was used to estimate the production rate of free light chains in multiple myeloma patients.

## Case studies

Here, various input-estimation approaches are illustrated using two case studies, fully specified in the supplement. Both optimal control-based and MCMC methods were implemented on top of CasADi, a framework for numeric optimisation [1]. This software can compute gradients and Hessians by automatic differentiation, and has interfaces to the ODE solver package SUNDIALS [21] together with the optimisation software IpOpt [38]. CasADi is implemented in C++, but at the time of writing, the recommended way to use it is to call it from Python.^1^ All the code for the case studies was written in Python 2.7, using CasADi version 2.4.1 for computing log posteriors, gradients and metric tensors as well as for performing optimisation with IpOpt. The code can be obtained at www2.warwick.ac.uk/fac/sci/eng/research/biomedical/impact/earlystageresearcher/magnustragardh/.

### Case Study 1

**Fig. 2:**
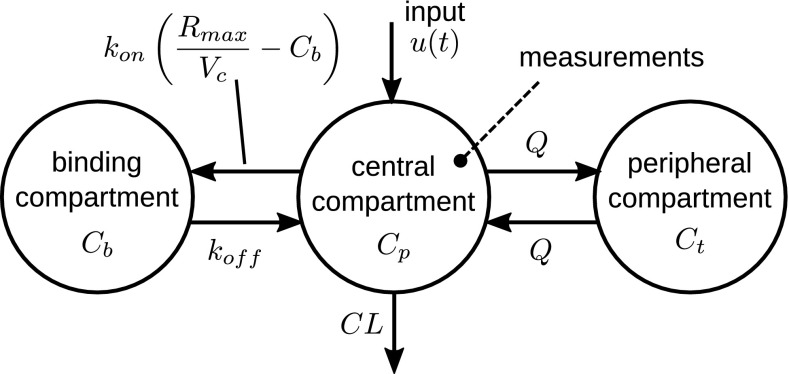
Three-compartment model for Case Study 1. The aim is to estimate the function *u*(*t*) given measurements of $C_p$

In this case study, input-estimation methods were applied to a dataset from a previously published study [22]. The purpose of the study was to characterise the pharmacokinetics of eflornithine, a drug used to treat Human African Trypanosomiasis. Using data from intravenous administration, a nonlinear 3-compartment model (Fig. 2) could be fitted to the data:

15 $$\begin{aligned} \frac{{\mathrm {d}}C_p}{{\mathrm {d}}t}\,=\, & {} \frac{u(t)}{V_c} - \left( \frac{CL}{V_c} + \frac{Q}{V_c}\right) C_p + \frac{Q}{V_c}C_t - k_{on} \cdot C_p \cdot \left( \frac{R_{max}}{V_c} - C_b\right) + k_{off} \cdot C_b \\ \end{aligned}$$

16 $$\begin{aligned} \frac{{\mathrm {d}}C_t}{{\mathrm {d}}t}\,=\, & {} \frac{Q}{V_t}C_p - \frac{Q}{V_t}C_t \end{aligned}$$

17 $$\begin{aligned} \frac{{\mathrm {d}}C_b}{{\mathrm {d}}t}\,=\, & {} k_{on}\cdot C_p \cdot \left( \frac{R_{max}}{V_c} - C_b\right) - k_{off} \cdot C_b \end{aligned}$$

where $C_p$, $C_t$ and $C_b$ are the drug concentrations in the central, peripheral and binding compartments, and $V_c$, $V_t$, *CL*, *Q*, $k_{on}$, $k_{off}$ and $R_{max}$ are parameters, defined in the original paper and in the supplement. The drug concentration in the central compartment $C_p$ was measured. When the drug is given intravenously, the input *u*(*t*) is an impulse. When the same drug is given orally, the input *u*(*t*) describes the absorption rate of the drug. Using the model and concentration data from experiments when the drug was administered orally, the absorption rate could be determined.

In the original article, this was done by fitting a smoothing spline to the concentration measurements, and directly inverting the system of ODEs. While this approach yielded satisfactory results, it is somewhat simplistic. Since it enforces smoothness on the output function rather than on the input, it is difficult to determine what assumptions about the input function are actually being made. Additionally, no attempt was made to assess estimation uncertainty.

For this paper, the input-estimation methods described previously were applied to two representative time series: one for a low dose of 20 mg/kg and one for a high dose of 1500 mg/kg.

**Fig. 3:**
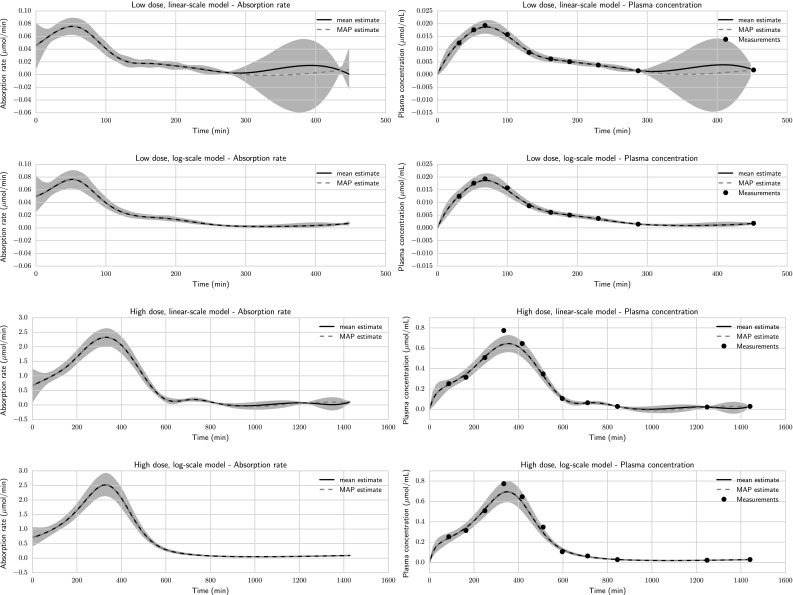
Estimated absorption rates, predicted plasma concentrations, and plasma concentration measurements for low and high doses with both log- and linear-scale models in Case Study 1. All estimates are calculated using the mid value for the regularisation parameter. The *shaded regions* are 95 % credible intervals. MAP estimates were obtained with single shooting, and mean and credible interval estimates were obtained with MCMC

Penalisation of the second derivative, as in Eq. (7) was chosen as a prior. Two input parametrisations were investigated: Cubic B-splines with breakpoints at the measurement times, and a piecewise constant function, discretised to 100 uniformly distributed intervals. Both MAP estimates and pointwise means and 95 % credible intervals were sought. To this end, optimal control methods as well as MCMC were used.

The time series are characterised by an early phase, where the plasma concentration has a large peak and the sampling is relatively dense, and a late phase, where the plasma concentration has declined and sampling is sparser (Fig. 3). To capture the initial peak, the amount of regularisation cannot be too large. On the other hand, too little regularisation can cause unrealistically large uncertainty in the latter sparsely sampled part. One way to mitigate this is to apply the prior to the logarithm of the input function rather than to the input function itself. This has the added benefit of automatically ensuring that the input function is always non-negative. In the sequel, this will be referred to as the “log-scale model”, while penalising the function itself will be referred to as the “linear-scale model”.

**Fig. 4:**
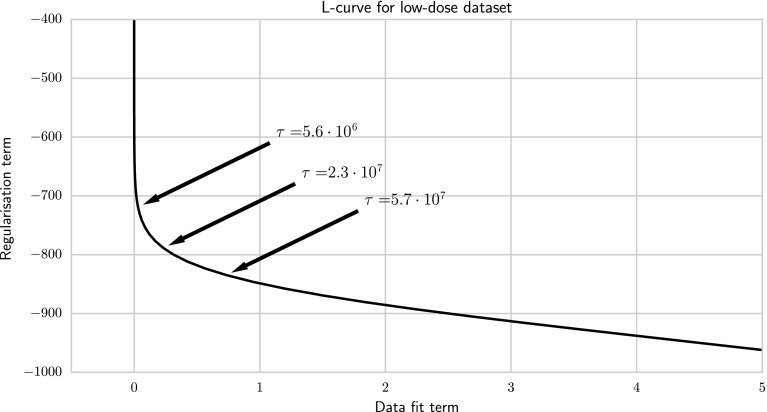
Example L-*curve* obtained for the low-dose dataset in Case Study 1. Three values for $\tau$ were selected to be used in the subsequent analysis

As a first step, a suitable value for the regularisation parameter $\tau$ was determined using the L-curve approach [18]. To investigate the sensitivity to $\tau$, the subsequent estimation was run using three different values: one at the “knee” of the curve, and one on either side of this. The resulting estimates were qualitatively similar for all three values. Fig. 4 shows a typical L-curve.

In summary, the following analyses were performed:

- MAP estimation using the cubic spline model.
- MAP estimation using the piecewise constant model.
- Full Bayesian estimation by MCMC using the cubic spline model.

All analyses were performed for both time series, and for both the linear- and the log-scale models. MCMC estimation turned out to be too inefficient when the number of parameters significantly exceeds the number of measurements. Therefore, MCMC estimation was not used for the piecewise constant model.

For the optimal control-based methods, single shooting was used for the spline model, since this made it easy to reuse the code for the MCMC estimation. For the piecewise constant model, single shooting was too inefficient and was replaced by collocation.

For MCMC, a simple component-wise Gaussian random-walk Metropolis–Hastings algorithm was used. In this method, each parameter is updated individually, using a Gaussian proposal density centred on the current value. The variance of the proposal density was tuned during trial runs to maintain the acceptance rate between 0.2 and 0.5, and hence keep it reasonably close to optimal values  [6, Sect. 4.2]. The tuning was performed by monitoring the acceptance rate every 100 iterations, and modifying the proposal variance if the acceptance rate was not in the range 0.2–0.5. The Markov chains were initialised to the MAP estimate given by the optimal-control techniques. 15,000 samples were drawn for each analysis.

Obtaining MAP estimates is in general computationally cheap. For the linear-scale model, even the 100-dimensional piecewise-constant model could be solved using collocation methods in a matter of seconds on an ordinary workstation. On the other hand, these methods were unable to find a solution to the log-scale version of the piecewise-constant model. Therefore, all results for the log-scale model were obtained using splines. MCMC is considerably more expensive—drawing 15,000 samples requires 20–30 min on the test machine. One reason for the long running time is that the parameters are updated one at a time, and the ODE solutions have to be recomputed for every update. Updating several parameters jointly would be more efficient, but finding good proposals for joint updates can be challenging. Methods for doing this are explored in Case Study 2.

The ESS, as defined in “Estimation Algorithms” section, was used as a measure of the quality of the samples. In particular, the ESS of the bioavailability was monitored, as it was considered to be the most important quantity. Typical ESS values ranged from 300 to 2000 samples. The notable exception was the linear-scale model with the low dose, where the quality of the samples from the Markov chain was very poor despite the large number of samples and extensive tuning. Here, the typical ESS was around 10 samples. The results from that analysis are therefore very uncertain.

From Fig. 3, it can be seen that for the high dose, the linear-scale model gives an underprediction of the initial peak at around 350 min. During 900–1400 min, the sparse sampling causes the uncertainty of the plasma concentration to drop below 0, clearly an unphysical result. The log-scale model appears to capture the peak better and keeps the uncertainty within reasonable values. For the low dose, the poor mixing of the Markov chain makes it hard to make any conclusions about the linear-scale model. The log-scale model appears to capture the data well.

**Fig. 5:**
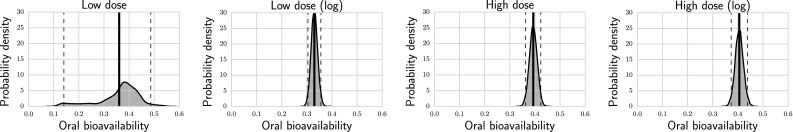
Kernel density estimate of the posterior of the oral bioavailabilities in Case Study 1. The *solid line* represents the mean, and the *dashed lines* show the 95 % credible interval

In Fig. 5, the bioavailability estimates are centred between 0.3 and 0.4. For comparison, in [22] the bioavailability was estimated to be 0.30 for the low dose and 0.38 for the high dose. The wide distribution of the linear-scale model with low dose can probably be attributed to the small ESS. For the high dose, the log-scale model predicts a slightly higher bioavailability than the linear-scale model. This is because the log-scale model is better at capturing the initial peak, which is the main contribution to bioavailability.

### Case Study 2

The data in this case study come from a drug-discovery project where the effect of two optimised monoclonal antibodies targeting fibroblast growth factor receptor (FGFR) 1c (*R1c mAb opt1* and *R1c mAb opt2*) was studied by measuring energy intake and body mass over time [11]. The parent R1c mAb has previously been shown to cause profound body weight and body fat loss due to decreased food intake (with energy expenditure unaltered), thereby improving glucose control in diet-induced obese (DIO) mice [23]. Thus, inhibiting R1c has become an attractive target for developing novel therapies against obesity and diabetes. However, different R1/R1c mAbs have been shown to decrease body weight solely due to hypophagia or via a combined effect on both food intake and energy expenditure [23, 36, 39], thus demonstrating the importance of taking both caloric intake and expenditure into account when defining mechanisms for weight-loss therapies. It is of great interest to be able to estimate energy intake without having to measure it directly, since methods for measuring energy intake can be unreliable, or expensive, or both [15]. The objective of this analysis was to investigate the possibility of estimating the energy intake from body-mass measurements alone.

The study consisted of seven groups of DIO mice: one vehicle group, and three groups of each of the two investigated substances, R1c mAb opt1 or R1c mAb opt2 administered as a single subcutaneous injection with doses of 0.3, 3 or 10 mg/kg. Each group comprised four mice, and energy intake was measured per group. Body mass was measured per individual, and group averages computed. Measurements were taken 9 days before treatment, and subsequently once per day or once every 2 days, up to 30 days after treatment. The analysis was performed using group means. All animal experiments were approved by the Gothenburg Ethics Committee for Experimental Animals.

The relationship between energy intake and body mass was characterised using the semi-mechanistic model of Guo and Hall [16, 17]. This model divides the total mass into fat mass (*FM*) and fat-free mass (*FFM*):

18 $$\begin{aligned} \frac{{\mathrm {d}}FFM}{{\mathrm {d}}t}\,=\, & {} \frac{\alpha }{\alpha \cdot \rho _{FFM} + \rho _{FM}} (EI - EE)\end{aligned}$$

19 $$\begin{aligned} \frac{{\mathrm {d}}FM}{{\mathrm {d}}t}\,=\, & {} \frac{1}{\alpha \cdot \rho _{FFM} + \rho _{FM}} (EI - EE) \end{aligned}$$

where *EI* is the energy intake, *EE* is the energy expenditure, $\rho _{FFM}$ and $\rho _{FM}$ are the densities of fat-free and fat tissue, and $\alpha$ is the *Forbes function*, relating changes in fat mass to changes in fat-free mass, empirically given by:

20 $$\begin{aligned} \alpha = q_1 + q_2 \cdot e^{q_3 \cdot FM} \end{aligned}$$

Furthermore, the energy expenditure is given by:

21 $$\begin{aligned} EE\,=\, & {} (K + \beta \Delta EI + (\gamma _{FFM} + \lambda )\cdot FFM + (\gamma _{FM} + \lambda )\cdot FM \\&+ \eta _{FFM}\cdot \alpha \cdot g \cdot EI + \eta _{FM} \cdot g \cdot EI)/(1 + \eta _{FM}\cdot g + \eta _{FFM}\cdot \alpha \cdot g) \end{aligned}$$

where *K* is a constant thermogenesis parameter, $\Delta EI$ is the difference between the energy intake and a standard reference intake (12 kcal/day), $\beta$ is a scaling parameter, $\eta _{FFM}$ and $\eta _{FM}$ are biochemical efficiencies for fat and protein synthesis, $\gamma _{FFM}$ and $\gamma _{FM}$ represent the relationship between metabolic rate and mass, and $g = 1/(\alpha \cdot \rho _{FFM} + \rho _{FM})$. The function $\lambda$ represents physical activity and could be captured by the empirical equation:

22 $$\begin{aligned} \lambda = {\left\{ \begin{array}{ll} \lambda _0 + \lambda _3 + \lambda _1\cdot \lambda _2\cdot t \cdot e^{-\lambda _2 t} &{} \text {if } t \ge 0 \\ \lambda _0 &{} \text {otherwise} \end{array}\right. } \end{aligned}$$

where $\lambda _i$, $i \in \{0, 1, 2, 3\}$ were estimated from data for each dose group and $t = 0$ is the start of the treatment. These parameters are not to be confused with the eigenvalues $\lambda _i$ in Eq. (11). All other model parameters were literature values, as used in [11] and defined in the supplement.

As choice of prior, the first derivative of the energy intake was penalised, which is equivalent to modelling the energy intake as a random walk. For the body-mass measurements, a proportional 0.5 % measurement noise was assumed.

The input functions were represented using Karhunen–Loève basis functions [28]. Twenty basis functions were used, as it was found that adding more did not significantly influence the estimates. A constant term, which was not penalised, was added to allow the energy intake to start at a nonzero value. The regularisation parameter was treated as an unknown parameter, and estimated jointly with the basis function coefficients. It was assigned a Gamma distribution prior, which is a conjugate prior to the inverse variance of the basis function coefficients. This makes it possible to estimate it using Gibbs sampling. The Gamma distribution was assigned a shape parameter of 0.001 and a rate parameter of 0.001, in order to make the prior flat and thus to avoid making strong *a priori* assumptions about the parameter value.

The sampling process was carried out as follows:

1. A good starting point for MCMC sampling was determined. This was done by fixing the regularisation parameter to a high value, and optimising the log posterior with respect to the basis coefficients.
2. MCMC sampling was done by alternating between the following updates:
  - Updating the regularisation parameter using Gibbs sampling.
  - Jointly updating the coefficients of the basis functions using SMMALA.

The computational bottleneck is the calculation of the Jacobian of the predicted body mass with respect to the basis coefficients, which is necessary in order to evaluate the metric tensor. To obtain an efficient algorithm, it is highly desirable to use sampling techniques which minimise the number of metric tensor evaluations. The sampling method used in 2(a) and 2(b) performs well since all basis coefficients are updated jointly, requiring only a single metric tensor computation per iteration. The regularisation parameter is cheap to update, since the log target and the metric tensor can be updated without recalculating the Jacobian.

**Fig. 6:**
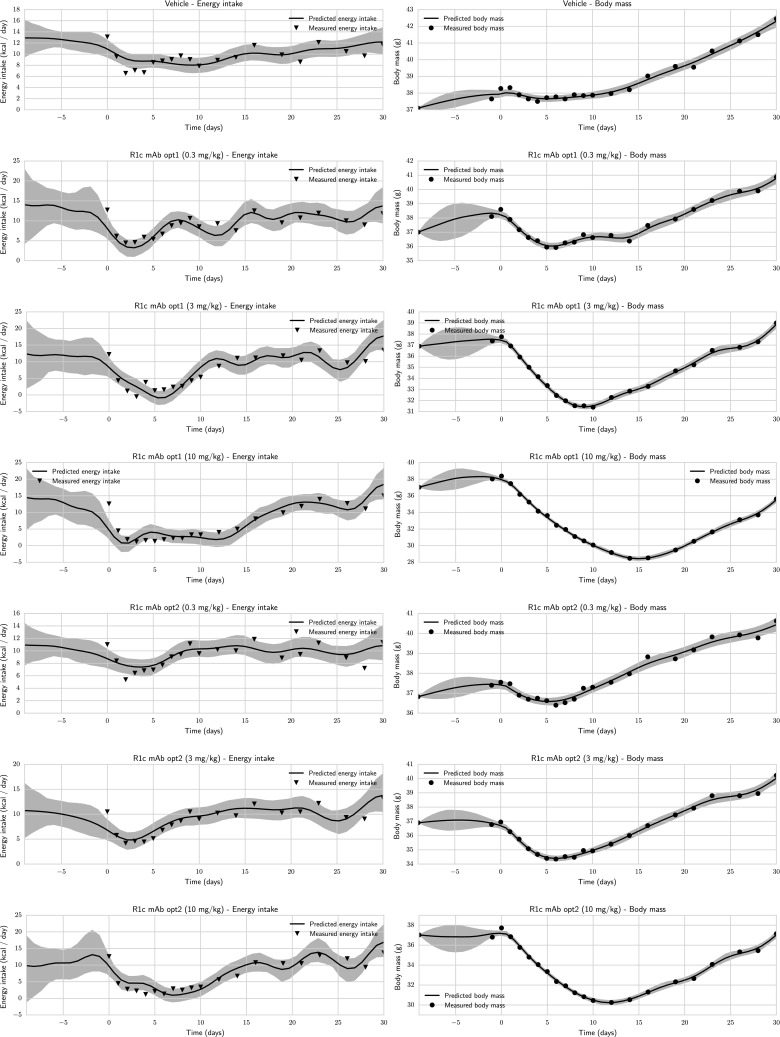
Measured and estimated energy intake and body mass for all datasets in Case Study 2. The body-mass measurements (*circles*) were used for estimation, while the energy-intake measurements (*triangles*) are not known to the estimation algorithm and are plotted for comparison with the estimates. The *shaded regions* are 95 % credible intervals

**Table 1:** Running time and ESS for the time-series in Case Study 2

Dose group	Method	Number of samples	Time (s)	Median ESS	Median ESS (s)
Vehicle	SMMALA	5000	151.1	940.8	6.2
R1c mAb opt1 (0.3 mg/kg)	SMMALA	5000	172.6	685.5	4.0
R1c mAb opt1 (3 mg/kg)	SMMALA	5000	179.1	601.6	3.4
R1c mAb opt1 (10 mg/kg)	SMMALA	5000	172.6	694.6	4.0
R1c mAb opt2 (0.3 mg/kg)	SMMALA	5000	151.6	866.8	5.7
R1c mAb opt2 (3 mg/kg)	SMMALA	5000	162.9	828.8	5.1
R1c mAb opt2 (10 mg/kg)	SMMALA	5000	173.4	551.0	3.2
R1c mAb opt1 (10 mg/kg)	RWMH	50,000	142.4	267.2	1.9

*ESS* effective sample size

In most of the measured time-series, the estimated inputs agree reasonably well with the data (Fig. 6). The main difficulty is the rapid decrease in energy intake seen around $t = 0$, which is hard to capture with a random-walk model. It can be noted that the uncertainty in the energy intake is considerably greater than in the body mass. This can be expected, since the model acts as a lowpass filter: rapid changes in energy intake do not cause equally rapid changes in body mass. There are therefore multiple energy intake profiles that would result in similar body mass measurements. A consequence of this is that fast oscillations are not captured reliably. Still, the method is honest in that it correctly gives an uncertainty region that includes most points. It can also be noted that the uncertainty in the energy intake as well as in the body mass is larger during 8 days prior to treatment where no measurements are taken. It is an attractive feature of these methods that such uncertainty is automatically accounted for.

**Fig. 7:**
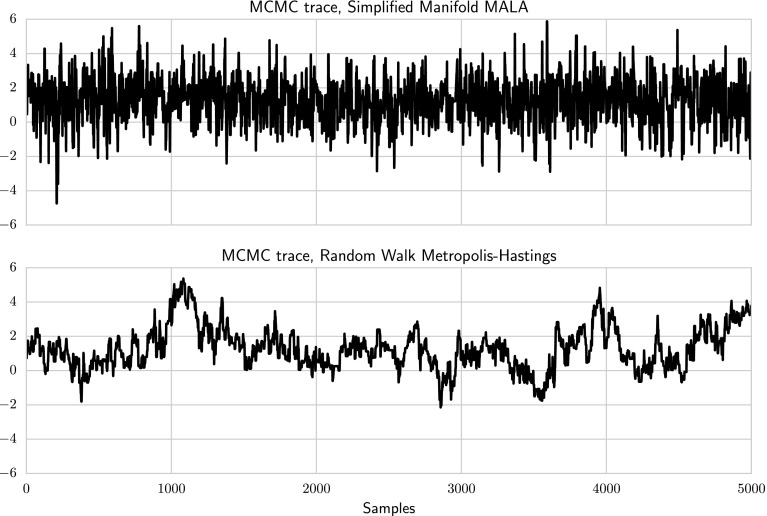
Representative MCMC traces for a parameter (R1c mAb opt1, 10 mg/kg, coefficient 6). It can be clearly seen that SMMALA traces explore the parameter space more efficiently

For comparison, a similar sampling scheme was tested on a single time-series, where the basis coefficients were jointly updated using the Random Walk Metropolis–Hastings (RWMH). The proposal covariance matrix was selected by computing the metric tensor at the MAP estimate, and scaling this to get an acceptance rate around 20–30 %. RWMH can draw samples approximately 10 times faster than SMMALA, since it does not need to evaluate the Jacobian. However, in terms of effective samples per second, SMMALA gives better performance, since its samples are considerably less correlated (Fig. 7). Furthermore, several trial runs had to be made to find a good scaling factor for RWMH, something which has to be done separately for each time-series. In contrast, SMMALA required no manual tuning. Running time and median effective sample size are shown in Table 1.

A weakness in the current work is that the measured energy intake was used to estimate the physical activity parameters $\lambda _i$, $i \in \{0, 1, 2, 3\}$. Since the purpose here was to evaluate the possibility of estimating the energy intake *given* that the system dynamics are known, this was considered acceptable. For the methods to be useful in experiments where the energy intake is not measured, it would be necessary to characterise the vehicle and drug effect on the physical activity by a generic model.

## Discussion

Both case studies show that, in terms of computational speed, MAP estimates can be obtained quickly for these kinds of models. Obtaining full posteriors in a reasonable amount of time is considerably more difficult. Case Study 1 shows that naive application of MCMC methods does not perform well in certain cases. SMMALA can be a good default choice, since it can efficiently update several parameters jointly, and does not require any user-specified tuning. It could also be worthwhile to investigate more advanced MCMC proposals, such as Hamiltonian Monte Carlo and Riemannian Manifold Hamiltonian Monte Carlo methods [14].

An alternative way to obtain estimates is to make multiple MAP estimates using bootstrapping. Note, however, that this will give a frequentist estimate of estimator variance rather than of uncertainty in the Bayesian sense. Great care has to be taken when interpreting and comparing uncertainty estimates from different methods.

It may also be worthwhile to investigate other parametrisations. Although the Karhunen–Loève expansion has appealing theoretical properties, it has the disadvantage that all basis functions are non-zero at all time points, making it necessary to sum all of them when evaluating at a single time point. They also make it difficult to impose non-negativity constraints.

The most principled and automated way to estimate the regularisation parameter is the Bayesian method of treating it as an additional random variable to be estimated from the data. However, for certain problems this method can have robustness issues. The L-curve method is an alternative, but it is far from ideal in terms of usability, since it requires the user to plot and manually select a value from the curve.

A disadvantage of using a linear-scale input model is that it does not rule out negative energy intakes. It is possible to impose non-negativity constraints by using a log-scale input model, as in Case Study 1. However, such a model may be hard to justify in certain cases, and it makes it more difficult to find efficient sampling methods. Another way to impose constraints would be to simply reject all proposed input functions that drop below zero. This could be reasonably efficient when only a small portion of the unconstrained distribution is below zero. In other cases, it could lead to prohibitively large rejection rates. A pragmatic approach is to use the unconstrained results as is, acknowledging that it is just an approximation. This is what has been done in Case Study 2.

In the case studies, uncertainties in the system model are not taken into account. Adding this could improve the statistical soundness and give more realistic estimates. This could be done by including uncertain system parameters in the estimation problem, estimating them jointly with the input function. Although it may be computationally expensive, it would not require any conceptual changes to the methods. It may also be possible to embed these methods in a non-linear mixed effects (NLME) framework.

CasADi has proved to be a valuable tool in that it allows the user to easily obtain gradients and Hessians for complicated functions, which can include calls to numerical ODE solvers. The details involved in formulating and solving the sensitivity equations are handled automatically by the software.

Since the performance of the methods may be problem-specific, it is important to evaluate them using multiple datasets and models. Only then can any conclusions about general usefulness be made.

## Conclusions and future work

Numerous drug discovery deconvolution-applications have been reported over the years, and it is evident that there is a need for useful methods also for the more general non-linear case, referred to as input estimation. This work serves as a highly promising starting point for application of input-estimation methods to problems in drug discovery: it gives a rigorous definition of the problem, it lists main methods and how these can be implemented, and discusses the application of the methods to realistic case studies. Additionally, the usefulness of CasADi for implementing these methods has been investigated and verified.

The presented methods for optimal control for input estimation can be recommended for use in drug discovery. The MCMC-based methods work well in certain cases, but they can have long running times, and care has to be taken to make sure that the parameter space is well explored. Further improvements would be desirable before they can be recommended for use by non-experts.

Suggestions for future work in this area include investigating the choice of prior and basis functions. It can be of interest to evaluate additional criteria for choosing the regularisation parameter. Note, however, that the regularisation parameter does not require any special treatment when Bayesian methods are used. Additionally, more advanced MCMC methods can be evaluated and alternative criteria can be considered for determining the number of MCMC samples necessary to obtain an accurate estimate.

To gain a better understanding of the performance of these methods, more combinations of prior choice, basis functions and estimation methods should be evaluated on a larger collection of input-estimation problems, ideally including both real and simulated data. The two fully specified problems of this paper form the base of such a collection. Desirable properties of input-estimation methods to evaluate include:

- Computational speed. The method should be fast enough to appeal to modellers working under time constraints.
- Usability. It should be possible for non-experts to use the method.
- Statistical soundness. All assumptions should be reasonable and explicitly stated. Sources of uncertainty should be accounted for.
- Usefulness. The algorithm should be applicable to as many input-estimation problems as possible.

## Electronic supplementary material

Supplementary material 1 (PDF 136 kb)

## Footnotes

1 www.python.org.
