# Supplementary material for: Input estimation for drug discovery using optimal control and Markov chain Monte Carlo approaches
Source: J Pharmacokinet Pharmacodyn. 2016 Mar 1;43:207–21. doi: 10.1007/s10928-016-9467-z (PMC4791487; doi:10.1007/s10928-016-9467-z)
Supplement: Supplementary file 1 — Supplementary material 1 (PDF 136 kb) [file 10928_2016_9467_MOESM1_ESM.pdf]

Supplement to "Input estimation for drug discovery using optimal control and Markov Chain Monte Carlo approaches"

Case study 1

Model

$$\begin{aligned}
\frac{\mathrm{d}C_p}{\mathrm{d}t} &= \frac{u(t)}{V_c} - \left(\frac{CL}{V_c} + \frac{Q}{V_c}\right) C_p + \frac{Q}{V_c} C_t - \\
&\quad k_{on} \cdot C_p \cdot \left(\frac{R_{max}}{V_c} - C_b\right) + k_{off} \cdot C_b \\
\frac{\mathrm{d}C_t}{\mathrm{d}t} &= \frac{Q}{V_t} C_p - \frac{Q}{V_t} C_t \\
\frac{\mathrm{d}C_b}{\mathrm{d}t} &= k_{on} \cdot C_p \cdot \left(\frac{R_{max}}{V_c} - C_b\right) - k_{off} \cdot C_b
\end{aligned}$$

Parameters

Parameter	Abbreviation	Unit	Value
Clearance	CL	mL/min	3.36
Central volume	V_c	mL	74.7
Peripheral volume	V_t	mL	31.6
Intercompartmental clearance	Q	mL/min	0.217
Total binding capacity	R_{max}	µmol	73.3
Binding rate constant	k_{on}	mL/min/µmol	0.00275
Dissociation rate constant	k_{off}	min^{-1}	0.000468

Data

**Dose 20 mg/kg, body mass 346.5 g**		**Dose 1500 mg/kg, body mass 362.4 g**	
Time (min)	C_p (µmol/mL)	Time (min)	C_p (µmol/mL)
31	0.0124	87	0.252
52	0.0176	164	0.314
68	0.0192	249	0.508
100	0.0157	334	0.774
131	0.00871	417	0.646
162	0.00614	511	0.348
189	0.00506	597	0.106
230	0.00374	711	0.0643
287	0.00150	845	0.0285
452	0.00182	1248	0.0225
		1441	0.0290

Case study 2

Model

$$
\begin{aligned}
\frac{\mathrm{d}FFM}{\mathrm{d}t} &= \frac{\alpha}{\alpha \cdot \rho_{FFM} + \rho_{FM}}(EI - EE) \\
\frac{\mathrm{d}FM}{\mathrm{d}t} &= \frac{1}{\alpha \cdot \rho_{FFM} + \rho_{FM}}(EI - EE) \\
EE &= (K + \beta \Delta EI + (\gamma_{FFM} + \lambda) \cdot FFM + (\gamma_{FM} + \lambda) \cdot FM \\
&\quad + \eta_{FFM} \cdot \alpha \cdot g \cdot EI + \eta_{FM} \cdot g \cdot EI) / \\
&\quad (1 + \eta_{FM} \cdot g + \eta_{FFM} \cdot \alpha \cdot g) \\
\alpha &= q_1 + q_2 \cdot e^{q_3 \cdot FM}
\end{aligned}
$$

Parameters

Parameter	Abbreviation	Unit	Value
Energy density, fat-free mass	ρ_{FFM}	kcal $\cdot$ g^{-1}	1.8
Energy density, fat mass	ρ_{FM}	kcal $\cdot$ g^{-1}	9.4
Metabolic rate, fat-free mass	γ_{FFM}	kcal $\cdot$ g^{-1} $\cdot$ day^{-1}	0.15
Metabolic rate, fat mass	γ_{FM}	kcal $\cdot$ g^{-1} $\cdot$ day^{-1}	0.03
Synthesis efficiency, fat-free mass	η_{FFM}	kcal $\cdot$ g^{-1}	0.23
Synthesis efficiency, fat mass	η_{FM}	kcal $\cdot$ g^{-1}	0.18
Thermogenesis rate	K	kcal $\cdot$ g^{-1}	2.1
Scaling factor, diet-induced thermogenesis	β	-	0.4
Standard energy intake	EI_{stand}	kcal $\cdot$ day^{-1}	12
Forbes function coefficient 1	q_1	-	0.13
Forbes function coefficient 2	q_2	-	0.02
Forbes function coefficient 3	q_3	g^{-1}	0.09

Physical activity parameters

Group	Parameter	Unit	Value
Vehicle	λ_0	kcal $\cdot$ day^{-1}	0.16
	λ_1	kcal $\cdot$ day^{-1}	0.18
	λ_2	day^{-1}	0.57
	λ_3	kcal $\cdot$ day^{-1}	-0.068
R1c mAb opt1 (0.3 mg/kg)	λ_0	kcal $\cdot$ day^{-1}	0.15
	λ_1	kcal $\cdot$ day^{-1}	0.25
	λ_2	day^{-1}	0.95
	λ_3	kcal $\cdot$ day^{-1}	-0.051
R1c mAb opt1 (3 mg/kg)	λ_0	kcal $\cdot$ day^{-1}	0.15
	λ_1	kcal $\cdot$ day^{-1}	0.54
	λ_2	day^{-1}	0.59
	λ_3	kcal $\cdot$ day^{-1}	-0.066
R1c mAb opt1 (10 mg/kg)	λ_0	kcal $\cdot$ day^{-1}	0.14
	λ_1	kcal $\cdot$ day^{-1}	0.52
	λ_2	day^{-1}	0.27
	λ_3	kcal $\cdot$ day^{-1}	-0.063
R1c mAb opt2 (0.3 mg/kg)	λ_0	kcal $\cdot$ day^{-1}	0.13
	λ_1	kcal $\cdot$ day^{-1}	0.21
	λ_2	day^{-1}	0.94
	λ_3	kcal $\cdot$ day^{-1}	-0.029
R1c mAb opt2 (3 mg/kg)	λ_0	kcal $\cdot$ day^{-1}	0.14
	λ_1	kcal $\cdot$ day^{-1}	0.33
	λ_2	day^{-1}	0.69
	λ_3	kcal $\cdot$ day^{-1}	-0.044
R1c mAb opt2 (10 mg/kg)	λ_0	kcal $\cdot$ day^{-1}	0.16
	λ_1	kcal $\cdot$ day^{-1}	0.59
	λ_2	day^{-1}	0.44
	λ_3	kcal $\cdot$ day^{-1}	-0.071

Data

Energy intake (kcal / day)

Time (days)	Vehicle	R1c mAb opt1 (0.3 mg/kg)	R1c mAb opt1 (3 mg/kg)	R1c mAb opt1 (10 mg/kg)
0	13.1	12.71	12.18	12.58
1	9.56	6.16	4.32	4.45
2	6.55	4.45	1.18	1.83
3	7.07	4.58	-0.52	1.18
4	6.68	5.9	3.8	1.57
5	8.52	5.37	1.31	1.31
6	8.78	6.68	1.57	1.83
7	9.04	8.78	2.36	2.23
8	9.69	9.43	2.62	2.23
9	9.04	10.61	4.32	3.28
10	7.86	8.52	5.37	3.28
12	8.91	9.3	8.65	4.0
14	9.43	7.53	11.07	4.98
16	11.59	12.51	11.13	8.06
19	9.87	9.52	11.79	9.91
21	8.58	10.74	10.48	11.86
23	12.12	11.92	13.3	13.95
26	10.48	10.0	9.69	12.71
28	9.69	8.97	10.09	11.07
30	11.66	11.79	13.43	15.0

Time (days)	R1c mAb opt2 (0.3 mg/kg)	R1c mAb opt2 (3 mg/kg)	R1c mAb opt2 (10 mg/kg)
0	11.0	10.48	12.58
1	8.38	5.76	4.45
2	5.37	4.19	2.75
3	6.42	4.58	2.23
4	6.81	4.45	1.18
5	6.94	5.11	2.1
6	7.73	6.81	1.31
7	9.04	7.86	2.88
8	9.43	8.65	2.49
9	11.13	10.48	3.14
10	9.56	9.04	3.41
12	10.22	10.22	5.63
14	10.02	9.69	6.68
16	11.86	12.05	10.74
19	8.86	10.26	10.48
21	9.43	10.48	10.48
23	11.27	12.18	12.97
26	8.91	9.34	11.92
28	7.21	9.04	9.37
30	11.33	13.3	13.69

Body mass (g)

Time (days)	Vehicle	R1c mAb opt1 (0.3 mg/kg)	R1c mAb opt1 (3 mg/kg)	R1c mAb opt1 (10 mg/kg)
-9	37.1	37.0	36.9	37.03
-1	37.65	38.1	37.38	38.02
0	38.28	38.6	37.75	38.38
1	38.33	37.9	36.92	37.48
2	37.9	37.17	35.92	36.17
3	37.65	36.63	35.0	35.25
4	37.5	36.4	34.15	34.15
5	37.73	35.95	33.35	33.63
6	37.78	35.93	32.45	32.45
7	37.65	36.25	31.98	31.95
8	37.9	36.3	31.53	31.1
9	37.85	36.83	31.52	30.58
10	37.88	36.63	31.4	30.07
12	37.98	36.77	32.28	29.18
14	38.2	36.38	32.85	28.48
16	39.02	37.48	33.27	28.53
19	39.6	37.92	34.67	29.48
21	39.55	38.6	35.22	30.52
23	40.52	39.23	36.52	31.65
26	41.13	39.88	36.8	33.13
28	41.5	39.9	37.3	33.7
30	42.45	40.88	39.0	35.63

Time (days)	R1c mAb opt2 (0.3 mg/kg)	R1c mAb opt2 (3 mg/kg)	R1c mAb opt2 (10 mg/kg)
-9	36.83	36.9	37.02
-1	37.4	36.78	36.8
0	37.55	36.95	37.73
1	37.48	36.27	36.85
2	36.9	35.75	35.77
3	36.7	35.08	34.8
4	36.75	34.67	34.05
5	36.63	34.4	33.35
6	36.4	34.35	32.35
7	36.53	34.53	31.93
8	36.7	34.48	31.23
9	37.25	34.95	30.82
10	37.3	34.92	30.45
12	37.55	35.4	30.25
14	37.98	36.0	30.52
16	38.83	36.7	31.3
19	38.73	37.45	32.32
21	39.17	37.92	32.65
23	39.82	38.8	34.08
26	39.92	38.8	35.33
28	39.77	38.95	35.45
30	40.63	40.23	37.15
